# Commentary: Association of the atherogenic index of plasma and high-sensitivity C-reactive protein with incident cardiovascular disease: evidence from a national cohort of middle-aged and older Chinese adults

**DOI:** 10.3389/fendo.2025.1684506

**Published:** 2025-10-10

**Authors:** XiaoMei Wang, SongQian Yang

**Affiliations:** Medical Laboratory Department, The First People’s Hospital of Honghe State, Gejiu, Honghe, Yunnan, China

**Keywords:** atherogenic index of plasma, C-reactive protein, cardiovascular disease, inflammation, commentary

Dear Editors,

We are writing to submit a Comment on the article entitled “Association of the atherogenic index of plasma and high-sensitivity C-reactive protein with incident cardiovascular disease: evidence from a national cohort of middle-aged and older Chinese adults” recently published in Frontiers in Endocrinology (16:1618157, 2025). This study makes a valuable contribution to understanding the joint role of lipid dysregulation (via AIP) and inflammation (via hs-CRP) in cardiovascular disease (CVD) risk among Chinese middle-aged and older adults, using a large nationally representative cohort. However, we wish to highlight several points that may enrich the interpretation of its findings and stimulate further discussion.

First, the study reports that the combined effect of elevated AIP and hs-CRP on CVD risk is not significant in individuals with prediabetes or diabetes, which contrasts with the strong association observed in those with normal glucose regulation. While the authors suggest potential explanations such as risk saturation or treatment effects, this finding warrants deeper exploration. For instance, individuals with abnormal glucose metabolism often exhibit complex metabolic disturbances (e.g., insulin resistance, altered lipoprotein metabolism) that may modify the interplay between AIP and hs-CRP. Further stratification by diabetes duration or medication type (e.g., statins, SGLT2 inhibitors) could clarify whether these factors blunt the observed associations. Additionally, the smaller sample size in the prediabetes/diabetes subgroups (n=1620 and 1403, respectively) may limit statistical power, and sensitivity analyses with expanded criteria for glucose abnormalities could strengthen this conclusion.

Second, the bidirectional mediation analyses reveal that hs-CRP mediates 6.6% of AIP’s association with CVD, while AIP mediates 20.3% of hs-CRP’s association. These relatively modest mediation proportions suggest that other unmeasured pathways likely contribute to the synergistic effects of lipid dysregulation and inflammation. Oxidative stress, a known link between lipids and inflammation in atherosclerosis, is mentioned in the discussion but not explicitly analyzed. Incorporating markers of oxidative stress (e.g., malondialdehyde, superoxide dismutase) in secondary analyses could help quantify their role as potential co-mediators, enhancing mechanistic insights. Similarly, exploring other inflammatory mediators (e.g., IL-6, TNF-α) alongside hs-CRP might reveal whether the observed effects are specific to CRP or reflective of a broader pro-inflammatory state.

Third, the predictive performance of the combined AIP-hs-CRP model, while superior to individual markers, remains modest (AUC = 0.590 for CVD, 0.615 for stroke). This aligns with the complex multifactorial nature of CVD but raises questions about clinical utility. The authors note that the model performs best in participants with BMI ≥28 kg/m², suggesting potential stratification by adiposity. It would be informative to explore whether integrating AIP and hs-CRP with traditional risk scores (e.g., China-PAR) improves reclassification metrics (e.g., NRI, IDI), as this would better demonstrate added value for risk assessment in clinical practice.

Finally, the study relies on self-reported CVD outcomes, which may introduce misclassification bias. While CHARLS is well-validated, supplementing with objective measures (e.g., hospital records, imaging data) where available could strengthen outcome ascertainment. Additionally, AIP is calculated using triglyceride and HDL-C levels, but recent evidence highlights the role of lipoprotein(a) or remnant cholesterol in CVD risk. Exploring interactions between these lipids and the AIP-hs-CRP axis could further refine risk stratification.

Overall, this study provides important epidemiological evidence for the integrated role of lipids and inflammation in CVD. We believe these considerations will help contextualize its findings and guide future research into targeted prevention strategies.

We confirm that this Comment has not been submitted or published elsewhere, and there are no conflicts of interest to declare.

Thank you for considering our contribution.

Sincerely,

